# Pathophysiology of Cerebral Malaria: Implications of MSCs as A Regenerative Medicinal Tool

**DOI:** 10.3390/bioengineering9060263

**Published:** 2022-06-20

**Authors:** Amrendra Chaudhary, Poonam Kataria, Neha Surela, Jyoti Das

**Affiliations:** 1 Parasite-Host Biology, National Institute of Malaria Research, New Delhi 110077, India; chaudharyamr@gmail.com (A.C.); punamkataria90@gmail.com (P.K.); neha.surela@gmail.com (N.S.); 2 AcSIR, Ghaziabad 201002, India

**Keywords:** cerebral malaria, pathophysiology, mesenchymal stem cells, regenerative medicine, endothelial cells, cytoadherence, blood-brain barrier breakage, platelet-mediated clumping, rosette formation, vascular edema

## Abstract

The severe form of malaria, i.e., cerebral malaria caused by *Plasmodium falciparum*, is a complex neurological syndrome. Surviving persons have a risk of behavioral difficulties, cognitive disorders, and epilepsy. Cerebral malaria is associated with multiple organ dysfunctions. The adhesion and accumulation of infected RBCs, platelets, and leucocytes (macrophages, CD4^+^ and CD8^+^ T cells, and monocytes) in the brain microvessels play an essential role in disease progression. Micro-vascular hindrance by coagulation and endothelial dysfunction contributes to neurological damage and the severity of the disease. Recent studies in human cerebral malaria and the murine model of cerebral malaria indicate that different pathogens as well as host-derived factors are involved in brain microvessel adhesion and coagulation that induces changes in vascular permeability and impairment of the blood-brain barrier. Efforts to alleviate blood-brain barrier dysfunction and de-sequestering of RBCs could serve as adjunct therapies. In this review, we briefly summarize the current understanding of the pathogenesis of cerebral malaria, the role of some factors (NK cells, platelet, ANG-2/ANG-1 ratio, and PfEMP1) in disease progression and various functions of Mesenchymal stem cells. This review also highlighted the implications of MSCs as a regenerative medicine.

## 1. Introduction

*Plasmodium falciparum* causes over 90 percent of all malaria infections. Children under the age of 5 years and pregnant women were the most susceptible groups affected by malaria. The World Health Organization (WHO) has characterized malaria as severe and uncomplicated. Delays in the detection and treatment of an uncomplicated infection of *P. falciparum* malaria lead to complications of severe cerebral malaria (CM). CM is usually caused by *P. falciparum*, but *Plasmodium vivax* is rarely responsible for CM complications. CM is a severe neurological complication caused by *Plasmodium falciparum* infection, resulting in high mortality rates. CM is characterized by brain tissue hemorrhage, the accumulation of infected red blood cells and mononuclear cells in brain microvessels, and blood-brain barrier (BBB) disruption. The precise mechanism of BBB disruption and brain injury is still unclear. Understanding the mechanism of brain injury and loss of the BBB is vital for further developments of therapeutic approaches [1]. CM is the leading cause of neural disability in children in the African region. Over 25% of children treated for cerebral malaria suffer from long-term neurocognitive impairment [2]. Main symptoms and risks include intracranial hypertension, hyperglycemia, repeated seizures, and deep coma. Altogether, these factors result in cerebral nervous system dysfunction and death [1]. The severity of cerebral malaria including mortality rate, depth of coma, and neurological symptoms in children differ from the adults. Moreover, intravascular hemolysis, hyperglycemia, and acidosis are common during cerebral malaria in children. On the other hand, cases of vital organ failure in children are less common than adults. Clinical reports suggest the involvement of acute renal failure in mortality; however, persisting renal failure is less reported in children. There have been 15–20% deaths reported in children even after the suggested treatment with anti-malarial drugs, i.e., cinchonoids or artemisinin derivatives. However, treatment with intravenous artesunate results in a lower mortality rate in adults [3].

The BBB separating brain parenchyma from peripheral blood is a semipermeable membrane. The binding of infected RBCs to the brain endothelium cells causes sequestering of infected RBCs in brain microvessels, leading to inflammation and cerebral swelling. Experiments confirm the presence of parasites within brain endothelial cells. The loss of the BBB facilitates *Plasmodium falciparum* plasma protein and fluid leakage into parenchymal and perivascular space, leading to cerebral vasogenic edema [4]. Electron microscopy reveals that BBB disruption results in parenchymal fluid increase and brain swelling [5].

Moreover, adhesion of infected RBCs to endothelial cells obstructs blood vessels, leading to sudden swelling in the brain and may result in death due to respiratory failure [4]. Although the sequestering of infected RBCs in the luminal site of brain microvessels is a keynote characteristic of human cerebral malaria (HCM) [6], only a few or no sequestrating of infected RBCs were observed in brain microvessels during experimental cerebral malaria (ECM) [7]. Moreover, no intracerebral accumulation of CD8^+^ T cells was observed during HCM [8]. There was a prominent pro-inflammatory cytokine response in the brain during ECM. Intracerebral accumulation of both CD8^+^ T cells was found essential for the development of ECM [9]. Moreover, infected RBCs were observed to be perivascular relative to several organs, followed by a morphological change in brain endothelial surface [10]. Targeting BBB disruption may be an optional therapeutic approach to reduce brain swelling and edema [5]. Moreover, the preventions of sequestration and de-sequestration of the infected RBCs are attractive therapeutic approaches.

## 2. Pathophysiology during Cerebral Malaria Progression

**Figure 1:**
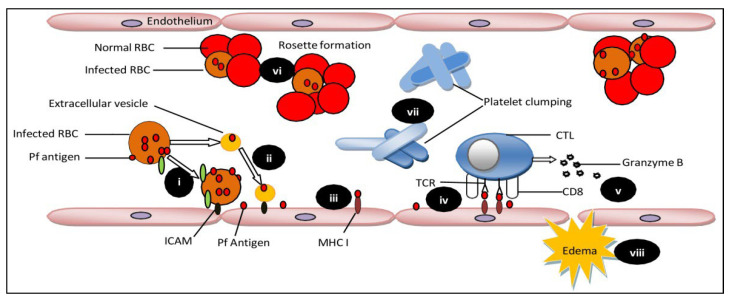
Factors involved in blood-brain barrier breakdown during cerebral malaria.

Several hypotheses have been reported to explain the mechanism of cerebral malaria development. One such hypothesis affirms that infected RBCs adherence to brain endothelium leads to blockage in microvessels, causing nutrient deprivation and hypoxia in nearby brain tissue. As a result, brain tissues cannot maintain membrane potential, which causes water inflow from extracellular to intracellular compartments, ultimately leading to cell death and tissue damage [11]. According to other groups, the sequestration of infected RBCs and inflammation enhance binding of infected RBCs with non-infected RBCs to form rosette [11] and the adherence of infected RBCs and leukocytes to brain endothelium (Figure 1) [12]. Moreover, platelet-mediated clumping of infected RBCs during infected RBCs sequestration [13] causes excess activation of the endothelial wall with pro-inflammatory mediators secretion [14] and an imbalanced release of endothelin-1 and angiopoietin-2 vasoactive mediators [15]. Finally, the brain endothelial cell barrier becomes impaired with leakage in brain parenchyma, resulting in breakage of the BBB.

In addition to acting as a transport system, endothelial cells also facilitate the binding of infected RBCs expressing *Plasmodium falciparum* erythrocyte membrane protein-1 (PfEMP1) linked with CM and act as phagocytic cells. Intercellular adhesion molecule (ICAM) dependent internalization of infected RBCs by endothelial cells results in brain injury and impairment of BBB [16]. After phagocytosis, infected RBCs undergo degradation and exposes host cells to cellular content and toxins [17]. ICAM-1 facilitates the entry of leukocytes in the endothelium by forming ring-like projections [18].

Transfer of Pf antigens from infected RBCs to brain endothelial cells by direct contact (i) or by production of antigen-carrying extracellular vesicles (ii); Pf antigen picked up and expressed by MHC I (iii); interaction of cytotoxic T lymphocytes (CTL) with endothelial cells expressing antigens, through their CD8 and TCR receptors (iv); granzyme B secreted by CTL (v); infected RBCs interact with uninfected RBCs leading to rosette formation (vi); platelet-mediated clumping (vii) disruption of the blood-brain barrier, leading to vasogenic edema (viii) (this figure is adopted and modified from Renia et al. (2020) [9].

In the case of infected RBCs, it is suggested that the mechanism is similar; however, infected RBCs show slower transit than leukocytes, as infected RBCs are deficient in machinery required for motility [18]. Preclinical studies showed that activated protein C (aPC), an anticoagulant serine protease, binds to endothelial protein C receptor (EPCR) and exerts its anti-inflammatory and anti-apoptotic activities, thereby protecting endothelial barrier functions [19,20]. EPCR, expressed at low levels by the brain endothelium, interact with infected RBCs and caused a loss of aPC, leading to a loss of cytoprotection [21].

**Figure 2:**
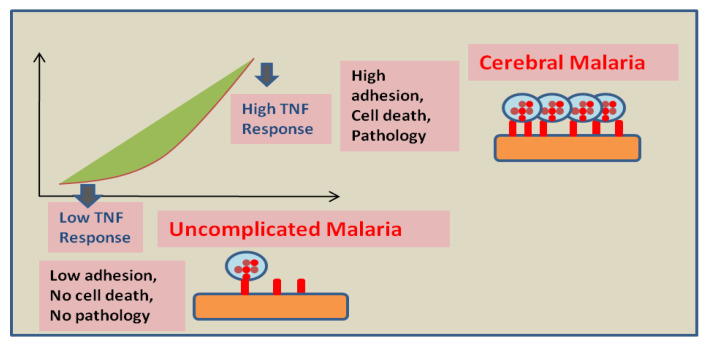
Proposed effect of the host endothelial response to TNF on disease severity: Low endothelium response to TNF leads to a minimal adhesion of infected RBCs and host cells and might be responsible for the absence of pathology. However, high endothelium response to TNF leads to elevated adhesion of infected RBCs. Moreover, a strong pro-apoptotic signal is generated in high responders that possibly results in BBB breakage and vasogenic edema (this figure is adopted and modified from Wassmer SC et al. [31]).

Consequently, infected RBCs binding with EPCR turn off cytoprotection via the CIDRα1 domain, whereas the ICAM-1 binding DBLβ domain incites binding and phagocytic response by the endothelium and assists cerebral malaria pathology through cerebral swelling and BBB disruption [16]. The link between ICAM-1 and EPCR to cerebral malaria symptoms has been established [16], but the mechanism of pathogenesis is still ambiguous. The activation of endothelial cells is regulated by cytokines such as Tumour Necrosis Factor alpha (TNF-α), Tumour Necrosis Factor beta (TNF-β), and interferon-γ [22]. The upregulation of TNF cytokine was observed in the blood and the brain cells of human and murine models of cerebral malaria [23]. In vitro TNF exposure induces the upregulation of infected RBCs adhesion factors and endothelial C activation markers, leading to increases in adherence of infected RBCs on human [24] or murine [25] brain microvascular cells (Figure 2). Furthermore, the upregulation of endothelial cells activation markers such as E-Selectin, von Willebrand Factor (vWF), ICAM-1, and CD36 all can act as adhesion targets for infected RBCs and contribute to endothelial activation.

Malaria parasite during infection suppresses erythropoiesis in the bone marrow, and hemozoin deposition in the tissues are mostly responsible for malarial anemia. Dyserythropoiesis results in the removal of infected and uninfected erythrocytes from the system and suppressed the production of mature erythrocytes in the bone marrow, which are primary contributors to the severity of malaria infection [26,27], followed by the development of severe malarial anemia leading to increased mortality and morbidity. The proliferation of erythroid progenitor cells is challenged during malaria infection due to decreased expression of erythroid-specific transcription factors such as GATA-1 and GATA-2 [28,29]. It has been reported that erythroblasts, BFU-E, and CFU-E numbers decrease after infection with *P. berghei* [30]. GATA-1 facilitates the survival and late-stage differentiation of BFU-E to CFU-E, which is facilitated by the mediator subunit MED1/TRAP220 [28,29], whereas GATA-2 is critical for the maintenance and proliferation of hematopoietic progenitors.

## 3. Platelets: A New Player in Infected RBCs Cytoadherence

Platelets are considered as critical contributors to CM by providing an alternate, indirect mechanism for infected RBCs adhesion [32]. Platelet sequestering observed only in CM brain and not in non CM brain [22]. Platelets accumulation were found colocalized with malarial pigments on the endothelial surface in Malawian patients died from CM, suggesting that the platelet closely interact with infected RBCs and endothelial cells during CM pathology [33]. Platelet-induced in vitro clumping experiments revealed the occurrence of infected RBCs clumping after incubation with Platelet-rich plasma. In the case of cerebral malaria, a high level of clumping was observed compared to uncomplicated malaria. However, in the presence of Platelet poor plasma, no clumping of infected RBC was observed in any of the patient groups after co-culture for 120 min [34].

Three platelet receptors CD36, globular C1q (gC1qR/HABP1/p32), and P-selectin have been identified for involvement in clumping [34,35,36]. CD36, a major receptor for infected RBCs, is constitutively express on platelets [37], suggesting that platelets could affect the adherence of infected RBCs on the brain endothelium, promoting vascular obstruction [38]. Both CD36 and P-selectin are known to be receptors for PfEMP-1 receptor of infected RBCs [39,40,41]. Infected RBCs are able to activate platelets [42] that lead to the transfer of the internal P-selectin to its surface [43]. Its expression on platelet membranes increased dramatically during clumping, confirming that infected RBCs activate platelets. Intercepting the clumping phenomenon using antibody-mediated blocking of CD36 and P-selectin encourages the idea of synergism between both receptors during clumping [34]. P-selectin increases the adherence of infected RBCs to CD36 on the endothelium [44,45] and may act as a trigger for the amplification of the clumping phenomenon. Platelets can form bridges between endothelial cells and infected RBCs [32,46,47] after bonding to the TNF activated endothelial cells with the help of specific molecules such as endothelial CD40 and platelet CD41. In addition to direct cytoadherence (infected RBCs to the endothelial wall), platelet-associated clumping and bridging may also contribute to the obstruction of the brain microvessels in CM patients.

Moreover, platelet activation leads to massive releases of transforming growth factor TGF-β1 from their a-granules [48]. Recent reports revealed that after amplified release and accumulation in brain microvessels, TGF-β1 might act together with TNF to induce endothelial apoptosis [14]. In addition, platelets are characterized as an important factor in assisting inflammatory reactions and immune responses [49]. The synchronized expression of adhesive and immune receptors on the platelet’s surface and release of some cytokines and inflammatory mediators can activate the interaction with leukocytes and increase their recruitment in brain microvessels. [50,51]. With the help of fibrinogen and ICAM-2, the IL-1β mediator can initiate the adhesion of leukocyte relative to endothelial cells [52,53]. Furthermore, IL-1β [54] can initiate leukocyte adhesion to endothelial cells that contribute to the obstruction of brain microvessels.

## 4. The Emerging Role of NK Cells in CM Pathogenesis

Natural killer (NK) cells are one of the earliest cells observed in the brain of *Plasmodium berghei* infected mice brain. In initial reports, no role of NK cells was found in ECM [55], but recent reports using anti-Asialo GM1 antibody confirms the active role of NK cells in ECM [56,57]. The antibody-mediated depletion of NK cells results in the inhibition of T cell recruitment in the infected brain of mice and confirms the role of NK cells in the regulation of adaptive immune response by modulation of the ability of T lymphocytes to migrate to the site of inflammation. [56]. T cells accumulation in brain microvessels plays a vital role in cerebral malaria pathogenesis [58]. The roles of CD4^+^ [55] and CD8^+^ [58,59] cells in disease assistance have been determined experimentally. Studies suggested that the sequestration of leukocytes and parasitized RBC in brain microvessels helps in disease induction [60,61]. Factors associated with the innate immune system are also involved in cerebral malaria induction. Based on the host’s genetic background, CD1d-restricted natural killer T (NKT) cells arbitrate resistance or susceptibility relative to cerebral malaria in mice [62].

Other than its pathological role, NK cells induce the production of IL-10 that inhibits pathological CD8^+^ cells response and protect against cerebral malaria in mice [63]. The disease model confirms the production of IL-10 by NK cells during infections [64,65,66]. A large quantity of interferon gamma (IFN-γ) is secreted by NK cells that play a critical role in immune-mediated brain damage in experimental cerebral malaria [67,68]. The treatment of brain endothelial cells of mice with IFN-γ upregulates the expression of intercellular adhesion molecule 1 (ICAM-1) and vascular cell adhesion molecule 1 (VCAM-1), which increases the adhesion of T lymphocytes by two- to three-folds [69]. Additionally, infected RBCs pretreated with IFN-γ showed significantly higher binding than that of untreated infected RBCs and normal RBCs.

## 5. Effect of Ang-2/Ang-1 Ratio in CM

Vascular endothelium activation and dysfunction are linked with the production of several biomarkers such as angiopoietin-1 (Ang-1) and angiopoietin-2 (Ang-2) in infectious diseases. The study on the role of Ang-1 and Ang-2 in endothelial cell serenity has become an essential and preferential topic lately [70]. Smooth muscle cells and pericytes continuously produce Ang-1, while endothelial cells produce Ang-2 that is stored in Weibel-Palade bodies. At the time of infection, Ang-2 is resealed rapidly from Weibel- Palade bodies [71]. Both Ang-1 and Ang-2 bind to the Tie-2 receptor, which is a member of the vascular tyrosine kinase receptor family. Under normal conditions, Ang-1 is present in high concentrations that allow it to bind with the Tie-2 receptor, leading to the activation of pro-survival pathways and inhibition of pro-inflammatory pathways. However, after infection, the rapid release of Ang-2 allows it to bind preferentially to tie-2 and promotes pro-inflammatory pathways [72]. A high concentration of plasma Ang-2 was observed in patients with severe malaria [72]. The plasma Ang-2/Ang-1 ratio is critical for endothelial activation, and it also act as a biomarker to differentiate uncomplicated malaria to CM. Elevated levels of Ang-2 are linked with mortality in patients with CM, while elevated levels of Ang-1 is related with uncomplicated malaria [73]. Since endothelial cell activation is a primary event during cerebral malaria, the biomarkers of the activated endothelium can be detected earliest before traditional disease markers and can be valuable biomarkers of disease severity.

## 6. Role of PfEMP1 in Infected RBCs Cytoadherence

The PfEMP1 is expressed on the membrane of infected RBCs and encourage the adherence of the infected RBCs on brain endothelial cells [74,75,76]. Unforeseen features of infected RBCs expressing PfEMP1 protein were discovered by a 3D spheroid model of the blood-brain barrier. The single haploid genome of *Plasmodium falciparum* encodes nearly 60 different PfEMP1variants, facilitating *Plasmodium falciparum* to undergo antigenic variation and dodges the immune system. PfEMP1 binds to a variety of receptors present on brain endothelial cells, including VCAM-1, cytokine-activated EPCR, and ICAM-1 [13,77,78], thereby fixing the infected RBCs on brain endothelial cells. Infected RBCs expressing PfEMP1 interact with ICAM-1 with their DBLβ motif [77]. Parasite-expressing PfEMP1 shows expansions in patient with cerebral malaria [77,79]. Moreover, the in vitro deletion of the DBLβ motif of PfEMP-1 results in a reduction in ICAM-1-specific adhesion of erythrocytes on the endothelium cells. Patients with high anti-PfEMP1 antibodies had a significantly reduced risk of developing symptomatic malaria [80]. After attachment to endothelium wall receptors, PfEMP1 may produce several signaling pathways that lead to restructuring tight junction complexes. Ultimately all these events result in impairment and breakage of the blood-brain barrier. Studies on leukocyte movement in brain endothelium, using murine and human models, suggest that the interaction of PfEMP1 and ICAM-1 promotes cytoskeleton-associated proteins phosphorylation, such as cortactin, paxillin, FAK, and p130Cas, that results in redesigning the endothelial cytoskeleton, which eventually assists in opening the blood-brain barrier [81].

## 7. Therapeutic Approaches

The sequestration of infected RBCs can also be prevented by using drugs targeting cytoadherence on the endothelium. In experimental cerebral malaria reports, the use of rapamycin restricts the cytoadherence of infected RBCs on endothelial cells by VCAM-1 and ICAM-1 reduction [82]. Moreover, the fungal ethanolic extract of *Trichoderma stromaticum* helps in reducing inflammation in experimental cerebral malaria by reducing the expression of VCAM-1 and ICAM-1, thus protecting the blood-brain barrier’s integrity [83].

The activation of endothelial cells by cytoadherence of infected RBCs and the pro-inflammatory effects of released cytokines play important roles in cerebral malaria pathophysiology [47]. Anti-TNF treatments can be helpful in managing the severity of disease and mortality rate. Endothelium activation triggers a TNF dependent pro-apoptotic pathway [84]. The difference in response of endothelial cells to TNF can affect the severity of the disease [85]. Anti-TNF therapy showed a positive result in vitro. However, it showed no reduction in mortality in the patients. Moreover, the use of antibodies against endothelial protein C receptor inhibited PfEMP1′s ability to bind to human endothelial cells [78]. Further studies on endothelial cells confirm the activation of Rac1 signaling via cross binding of VCAM-1 that results in Rho-dependent inductions of stress fibers, leading to the weakening of tight junctions [81].

Moreover, the interaction of EPCR with activated protein C is prevented by binding PfEMP1 to EPCR. This binding shows two impacts: (i) promoting the activation of tissue factors Va and VIIIa, which results in the disablement of the aPC-mediated anti-coagulative pathway. These factors are responsible for thrombin generation on activation, which eventually results in fibrin deposition. (ii) The initiation of NF-kB and Rho A pathways through thrombin-mediated scissions of PAR1 is also observed. The activation of these pathways generates a pro-inflammatory response, which leads to impairment and breakage of the blood-brain barrier [20,79,86]. The administration of aPC may prevent blood-brain barrier dysfunction by inhibiting thrombin activity [87]. Other approaches embrace the utilization of endothelial cells isolated from patients suffering with cerebral malaria. Moreover, measuring the consequences of Ang-1 on endothelial cells pre-treated with TNF will be crucial for the development of new additional therapies and improving disease outcomes in CM. Since Ang-1 acts as a serenity agent for endothelial cells in the exquisite model of sepsis [88], maintaining a higher Ang-1concetration in Ang-2/Ang-1 ratio during infection can potentially block and reverse ongoing inflammatory processes in CM patients.

## 8. MSCs as A Regenerative Therapy

### 8.1. MSCs Mediated Cellular Mechanism of Protection

Mesenchymal stem cells (MSCs) first observed in the bone marrow are multipotent cells [89], which can differentiate into various cell lines lineages such as adipocytes, chondrocytes, and osteoblasts and display strong tissue protective and restorative properties. During malaria infection, MSCs become accumulated in primary and secondary lymphoid organs. Different surface antigens are expressed by MSCs in different tissues, which facilitate tissue repair and regeneration through the secretion of various soluble factors. Souza MC et al. demonstrated that, in *P. berghei*-infected mice, neurons were damaged, with an increased number of astrocytes and oligodendrocytes. However, in *P. berghei*-infected mice treated with BM-MSCs, brain damage was repaired, which leads to a reduction in parasitemia and mortality [90]. There was a substantial increase in phagocytic neutrophils in the brain [91]; hepatocytes and Kupffer cell regeneration in MSC-infused mice indicate regenerative ability of MSCs. MSCs promote hematopoiesis by releasing different critical molecules involved in the self-renewal, proliferation, and differentiation of hematopoietic stem and progenitor cells (HSPCs). Other than that, MSCs encourage the formation of colony-forming units-erythroid (CFU-E) cells in the bone marrow [92], which ultimately helps in preventing malarial anemia. These observations suggest that cell-based therapeutics for intervention in malaria may be useful in achieving sterile clearance and preventing disease reactivation.

### 8.2. MSCs Mediated Molecular Aspects of Protection

Studies on anti -CD3^−^, -CD19^−^, -Sca-1^+^, and -CD34^+^ antibodies revealed a significant elevation in Sca- 1^+^ and CD34^+^ cells in the lymph node and spleen of MSC-treated mice as compared to untreated mice [91]. A drop in GATA-1 and GATA-2 expression was observed in plasmodium-infected mice. Since GATA-1 is a key erythroid cell differentiation factor, the low level of GATA-1 expression may be responsible for reduced RBC formation in malaria-infected animals. However, after MSCs treatment, an increase in the expression of GATA-1 and GATA-2 has been reported in animals infused with MSCs. Increased CFU-E formation and reduced hemozoin content in MSC-infused mice support the conclusion that MSCs elicits signals to support erythropoiesis [93].

### 8.3. Application of MSCs in Animal Model of Cerebral Malaria

Malaria infection causes a reduction in CD4^+^ T cells in mice models of cerebral malaria, which are crucial for immunity development against malaria, leading to impaired T cell-mediated immunity [94]. However, an increase in the number of CD4^+^ and CD8^+^ T cells was reported in MSC-infused mice, and MSCs are able to rescue the proliferation of CD4^+^ T cells [92]. Ongoing research studies are focused on signaling molecules engaged in restoring immune responses by MSCs. MSCs also inhibited the induction of the negative co-stimulatory receptor programmed death-1 by T cells in recipient animals. Taken together, MSCs help in the protection against malaria infection by reprogramming hematopoiesis, by enhancing the differentiation of CD34^+^ cells, restoring CD4^+^ and CD8^+^ T cell proliferation, and by suppressing the expression of negative co-stimulators on T cells. The increased production of interleukin IL-12, which is crucial for self-renewal and differentiation of multipotent progenitor cells and suppressed production of IL-10, was reported in MSC infused animals. The transfer of MSCs isolated from secondary lymphoid organs of *P. berghei*-infected mice conferred host resistance against malaria through the enhanced production of pro-inflammatory cytokines IL-6, IL-12, and TNF-α, and the suppression of IL-10 [93,95].

Preclinical studies on several metabolic disorders, tissue regeneration, cancer, heart disorders, and other disorders suggest the robust use of MSC-based therapy [96]. Despite multiple preclinical studies on MSC-based therapy, no promising clinical studies are available for cerebral malaria. The multipotent nature and the potential of MSCs of modifying the tissue microenvironment makes them appropriate candidates for the development of stem cell-based regenerative medicinal therapy.

## 9. Conclusions

Current therapeutic approaches have been unsuccessful to prevent neuro-cognitive complications and deaths in cerebral malaria cases. Stem cell therapy is a newly emerging treatment for a wide range of disorders. A number of studies have pointed out the importance of MSC-based treatments in many disease conditions such as cancer, Parkinson’s disease, Huntington’s disease, transplantation, etc. Reports of experimental cerebral malaria suggest that bone marrow-derived MSC treatment reduces mortality and parasitemia; hemozoin accumulation deposition in the spleen, liver, kidney, and lung; and increases survival [90]. An appropriate evaluation of both preclinical clinical and clinical efficacy and the analysis of mechanisms by which MSCs protect the host against malaria should be performed. A further understanding of the immune-modulatory properties and impact of MSCs on erythropoiesis would make it possible to establish MSCs as a novel regenerative medicine in malaria research.
